# Are clinical risk scores for COPD useful?

**DOI:** 10.1136/bmjresp-2014-000072

**Published:** 2015-04-01

**Authors:** Jennifer K Quint

**Affiliations:** Department of Non-Communicable Disease Epidemiology, London School of Hygiene and Tropical Medicine, London, UK

**Keywords:** COPD epidemiology

Chronic obstructive pulmonary disease (COPD) is the second most common cause of emergency admission to hospital in the UK and one of the most costly inpatient conditions treated by the National Health Service (NHS).^1^ One in eight people over the age of 35 has undiagnosed COPD and, in recent years, there have been public health campaigns to identify those ‘missing millions’ in the UK.^2^ COPD is a diagnosis based on clinical symptoms, confirmed by the presence of obstructive spirometry^3^ and there is unreliable evidence for the initiation of large scale screening approaches to identify individuals at high risk. Therefore, a mechanism by which individuals can easily be identified as being high risk, from general practice (GP) databases, and be invited for spirometry, has important public health implications.

Electronic health records (EHR) are an increasingly popular resource in which to conduct research. Owing to the large volume of patients encompassed, they provide tremendous statistical power to answer many clinical questions. However, the devil is in the detail, as the outcomes obtained from this type of research are only as good as the methods used to identify those outcomes to begin with. A recent paper by Jones *et al*^4^ highlighted the very important issue of missed opportunities for identifying individuals with COPD at an earlier point in time in primary care. However, in analysing these data, the authors did not appreciate the fact that Read codes used within GP databases change over time. Some of the codes in the code list they used to identify people with COPD did not exist at the time they attributed a GP to have missed an opportunity to make a COPD diagnosis. As a result, the number of diagnostic opportunities missed was overestimated.

In this issue, Haroon *et al*^5^ have published a case–control study undertaken between 2000 and 2006 in which they have developed and validated a clinical risk score for use in primary care to identify people at risk of COPD using data from the Clinical Practice Research Datalink (CPRD). The clinical score incorporates smoking status, a previous diagnosis of asthma, lower respiratory tract infections (LRTI) and salbutamol prescriptions in the previous 3 years. The score developed, when applied to patients over the age of 35 years and used at their suggested threshold of 2.5, would have a positive predictive value of 22.6%, a negative predictive value of 97.6% and an overall screening yield of 3.5% for identifying people with COPD. In real terms, for every 29 records screened, 5 patients would require a clinical assessment to identify one patient with COPD. Development of clinical risk scores is important for population level screening, and the authors of this paper should be commended for their statistical rigour and the clarity of their methodology. However, there are several important caveats, which require the reader to be cautious in interpreting the results.

First, cases were identified between 2000 and 2006, spanning the implementation of the first quality and outcomes framework (QOF) for COPD in 2004,^6^ from which point the accuracy of coding associated with a COPD diagnosis improved significantly. While the authors acknowledge one of the weaknesses of their paper is not using a validated COPD codelist, thus bringing the accuracy of the diagnosis of COPD into question, this unfortunately leaves the reader questioning whether the outcome of this paper is actually just a score that predicts which patients will be mislabelled as having COPD in the future. While validation studies are laborious, they are crucial in ensuring accuracy of interrogation of EHR; use of a validated COPD definition would have strengthened this paper.^7^

Second, smoking is the predominant risk factor for COPD in the UK. Within EHR, recording of smoking is not always complete or accurate, particularly pre 2004. In this study, nearly 20% of the cases had never smoked, calling into question the accuracy of defining the cases. Likewise, misclassification of the controls may also have occurred, as the controls who smoked and had symptoms may well have had COPD and not had spirometry. How this affects the clinical risk tool will depend on the extent to which any misclassification is differential.

Third, owing to the fluctuating nature of coding, the lack of longevity of specific Read codes, the introduction of new Read codes and new coding systems being introduced (such as SNOMED-CT), tools such as this have a limited lifetime of usage. Furthermore, with changes in clinical practice and updates to QOF, this algorithm would benefit from validation in a more recent data set. Finally, the number and timing of several of the variables used in the score are likely to affect accuracy. For example, a patient's risk of COPD is likely to be different if they have had one LRTI in the proceeding 3 years as opposed to more.

Certainly what this paper does provide is an excellent preliminary analysis from which future models could be developed, but this risk score in its current format is by no means a definitive tool. Undoubtedly, a validation tool that could be incorporated into GP software to identify people at risk of COPD would be extremely useful.
